# Acute Œdema of the Tongue

**Published:** 1902-03

**Authors:** Orr Graham

**Affiliations:** Port Perry, Ont.


### ACUTE ŒDEMA OF THE TONGUE.

BY ORR GRAHAM, V.S.,
PORT PERRY, ONT.

THE subject of my paper is one of peculiar interest to me from a practical point of view, and, so far as I have been privileged to read in our scientific works, I have not at present any recollections or record of a similar affection, and although glossitis might be very easily associated in some particulars, yet I claim considerable difference does exist between the two affections.

The name given to the affections by myself (acute œdema of the tongue) does not throw any light upon the subject, yet I have no doubt but many other practitioners have encountered many and similar cases heretofore, and if not, then this paper will be of more or less importance to you; and whatever the primary or premonitory manifestations may have been I am unable to relate, in so far as the owners were not themselves able to notice any change prior to the commencement of the swelling or arrest of proper mastication, which apparently was of rapid or acute origin in each and every instance. As near as I can remember, they were all horses that were under proper care and feed or at work, in good condition and well cared for, nor had they been subjected to any unusual conditions prior to or at time of attack.

Symptoms developed were as follows: General and rapid swelling of the tongue, until in every instance it completely filled the cavity of the mouth to such an extent that the jaws were pressed apart as wide as the muscles of the cheeks would admit and the tongue protruded out some distance, and always accompanied by a profuse and an abundant discharge of a jelly-like fluid in consistency, yellowish in color when coming from the nasal cavities, but not so strong in color from the mouth—probably due in color to the different membranes from which it was secreted. In some cases the head and lips participated in the swelling more or less. General arrest of the natural excretions, from the effect that the affection had upon the general system. Great difficulty in respiration, according to the quantity of fluid accumulated in the nostrils; but if not overabundant in amount in the nasal chambers, then not much stress in respiration, showing that the œdema was not extensive in the epiglottis. The tongue did not appear to be sensitive where pressure was brought to bear upon it. In the more severe cases there were movements of restlessness, which naturally precedes approaching asphyxia or suffocation. A haggard and anxious expression was quite noticeable in most cases. The above symptoms were fully developed in less than twenty hours from the first appearance of the attack. It was not my privilege to see any of them until symptoms were well developed.

*Treatment.* First cleanse mouth out with warm water containing liquor ferri perchloride, to remove the abundant secretions. Do this before giving a purge of aloes in solution, which is attended by some difficulty; but by well elevating the head and exercising patience and careful attention the solution will gravitate into the throat, from which the animal can swallow it. Give injections to relieve bowels and hasten action of purge; constant and continued fomentations to the swollen lips and tongue, with warm water, to which add some astringent—I prefer liquid ferri perchloride from past experience. It is generally necessary to tie the head well up by two tie straps, thereby giving the circulation a much better chance to aid in recovery, arranging some high construction upon which to place your bath-tub, so as to be the more effectual, the patient receiving the full benefit of the heat as well as the application. I have not seen sufficient benefit derived from scarifications to warrant approval in those cases, neither do I condemn such. Keep the nasal chambers clear as possible of the abundant and thick accumulation, which, in some cases, will admit of being drawn out very lengthy before parting. Encourage action of the kidneys by nitrate of potash, and aid in correcting mucous membranes by soda bicarb. and vegetable tonics, giving drink in sufficient quantities as soon as circumstances will permit. The most of the cases respond favorably to treatment if taken in reasonable time ; once the swelling starts to recede, recovery is quite speedy and permanent, the animal being again ready for work in a few days. I have had but two cases die, one of them in about twenty hours from commencement of attack ; and so acute was the attack and so abundant and thick the accumulation collecting in the mouth, and more particularly in the nasal cavities, that suffocation could not be averted by the appliances I had at my disposal, being some fifteen miles from home and without a tracheotomy tube.

---

### THE HORSE.

SOCIETY owes to the horse a debt of gratitude a thousand times greater than it does to thousands of men who abuse him. He has ministered to progress ; has made social intercourse possible when otherwise it would have been slow and occasional or altogether impossible. He has virtually extended the strength of man, augmented his speed, doubled his time, decreased his burdens, and, becoming his slave, has relieved him from drudgery and made him free. For love's sake, for the sake of social life, for eminent moral reasons, the horse deserves to be bred, trained, and cared for with scrupulous care. The teaching of men how to do it has been left too long to those who look upon the horse as an instrument of gambling gains or of mere physical pleasure.

---

IT is gratifying to note frequently in the advertisements of noted breeding farms of thoroughbred cattle that all their animals are tuberculin tested, some going so far as to name the veterinarian who conducts this work.

---

WEISENBURG, Ontario, reports a virulent outbreak of anthrax that has caused the death of one veterinarian and a large number of cattle. It has prevailed at certain periods yearly for ten years. Its true nature not being recognized, the improper disposition of the carcasses have contributed to the maintenance of the disease on this farm.

---

THE activity of the veterinarians in Ohio in invoking the law against illegal practitioners has resulted in finding a great many other violators and suggested their removal to safer fields.

